# Immunocomplexed Antigen Capture and Identification by Native Top-Down Mass Spectrometry

**DOI:** 10.1021/jasms.3c00235

**Published:** 2023-09-08

**Authors:** John P. McGee, Rafael D. Melani, Ben Des Soye, Derek Croote, Valerie Winton, Stephen R. Quake, Jared O. Kafader, Neil L. Kelleher

**Affiliations:** † Departments of Chemistry and Molecular Biosciences and the Proteomics Center of Excellence, Northwestern University, Evanston, Illinois 60208, United States; ‡ Department of Bioengineering, Stanford University, Stanford, California 94305, United States; § Department of Applied Physics, Stanford University, Stanford, California 94305, United States

**Keywords:** native, antibody, antigen, Orbitrap, complex-up, top-down

## Abstract

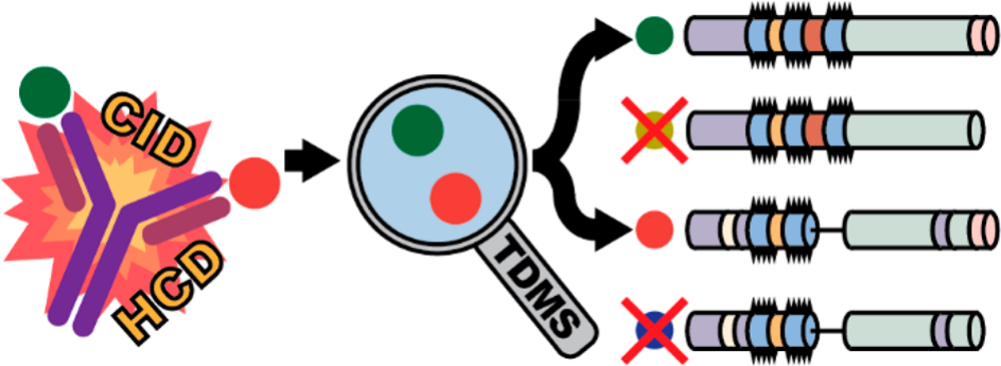

Antibody–antigen interactions are central to the immune response. Variation of protein antigens such as isoforms and post-translational modifications can alter their antibody binding sites. To directly connect the recognition of protein antigens with their molecular composition, we probed antibody–antigen complexes by using native tandem mass spectrometry. Specifically, we characterized the prominent peanut allergen Ara h 2 and a convergent IgE variable region discovered in patients who are allergic to peanuts. In addition to measuring the antigen-induced dimerization of IgE antibodies, we demonstrated how immunocomplexes can be isolated in the gas phase and activated to eject, identify, and characterize proteoforms of their bound antigens. Using tandem experiments, we isolated the ejected antigens and then fragmented them to identify their chemical composition. These results establish native top-down mass spectrometry as a viable platform for precise and thorough characterization of immunocomplexes to relate structure to function and enable the discovery of antigen proteoforms and their binding sites.

## Introduction

Proteoforms are the many protein end products originating from a single gene, considering their modifications, sequence permutations, and isoform variants.^1^ As such sources of variation can alter both protein function and their antibody binding sites, understanding the unique composition of proteoforms is crucial in understanding function and molecular recognition in biology.^2^ To this end, top-down mass spectrometry (TDMS) can systematically discover intact proteoforms by asserting the coexistence of modifications on distinct isoforms as they exist in biomolecular complexes.^3^ Characterization starts by isolating the ionized target based on its mass-to-charge ratio (*m*/*z*). The covalent bonds of isolates are then fragmented, usually through collisions with neutral gas, electron capture, or a combination of these activation methods.^4,5^ Comparisons of observed and theoretical fragment ion masses then identify the species and localize the placement of various modifications within the full-length protein sequence.

When TDMS is coupled to immunoprecipitation enrichment (immunoprecipitation mass spectrometry, or IP-MS), this workflow identifies proteoforms after elution off the antibody.^6^ However, IP elution typically denatures noncovalent complexes and dissociates bound metals and cofactors, which means that neither the immunocomplex nor any multiproteoform complexes enriched as antigens can be directly identified. Alternatively, “native” MS preserves endogenous noncovalent interactions for characterization, simply by using buffer solutions at neutral pH containing little or no organic solvents.^7^ While there is substantial precedent in the literature for native MS characterization of noncovalent immunocomplexes,^8−10^ there is limited literature on antigen ejection as a consequence of activation in the gas phase (“antigen ejection”).^11^ Furthermore, the cited example of antigen ejection was conducted at low resolution and stopped short of characterizing and confirming the antigen’s identity through fragmentation. Another work, by Zhang *et al.*, showcases direct fragmentation of an immunocomplex (using only the fragment antigen binding region as opposed to the entire antibody), introducing ambiguity in cases where multiple antigens are bound.^12^

**Figure 1:**
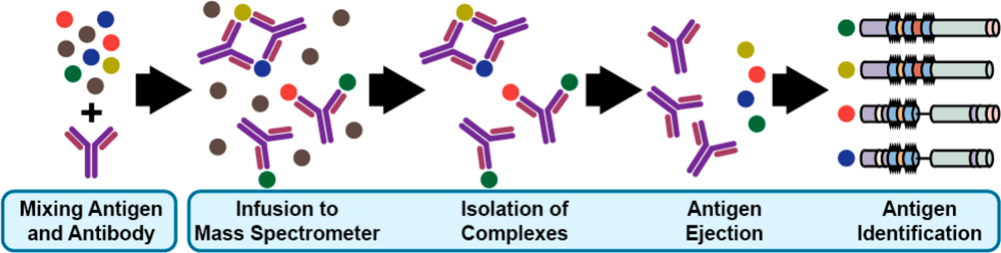
After the antibody and the antigen are mixed (far left), native electrospray infuses complexes into the mass spectrometer. Particles not bound to the antibody can be filtered away on the basis of *m/z*, and the complex can be activated to eject the bound antigen. Free antigens can then be targeted for profiling and deep characterization, revealing the presence of isoforms and location of modifications.

Here we put forth the first demonstration of proteoform-specific antigen identification from immunocomplexes via native TDMS, a maturation of concepts introduced and explored in the works referenced above. We analyzed the interactions between the four major isoforms^13^ of peanut allergen Ara h 2 and the convergently evolved variable region of an IgE, with picomolar binding affinity for Ara h 2, found in a clonal family of six plasmablasts from two unrelated peanut-allergic patients.^14^ As depicted in Figure 1, the immunocomplex and its components were analyzed together to create an unambiguous link between the immune response and specific proteoforms of bound antigens, including their binding sites. Bound complexes are then isolated by virtue of their mass-to-charge ratio and activated to eject, partially sequence, and unambiguously identify the antigen(s) recognized by the recombinant antibody. When extended more broadly, this approach could have significant impact in identifying “orphan” antigens like those encountered in autoimmune disorders.

## Results

**Figure 2:**
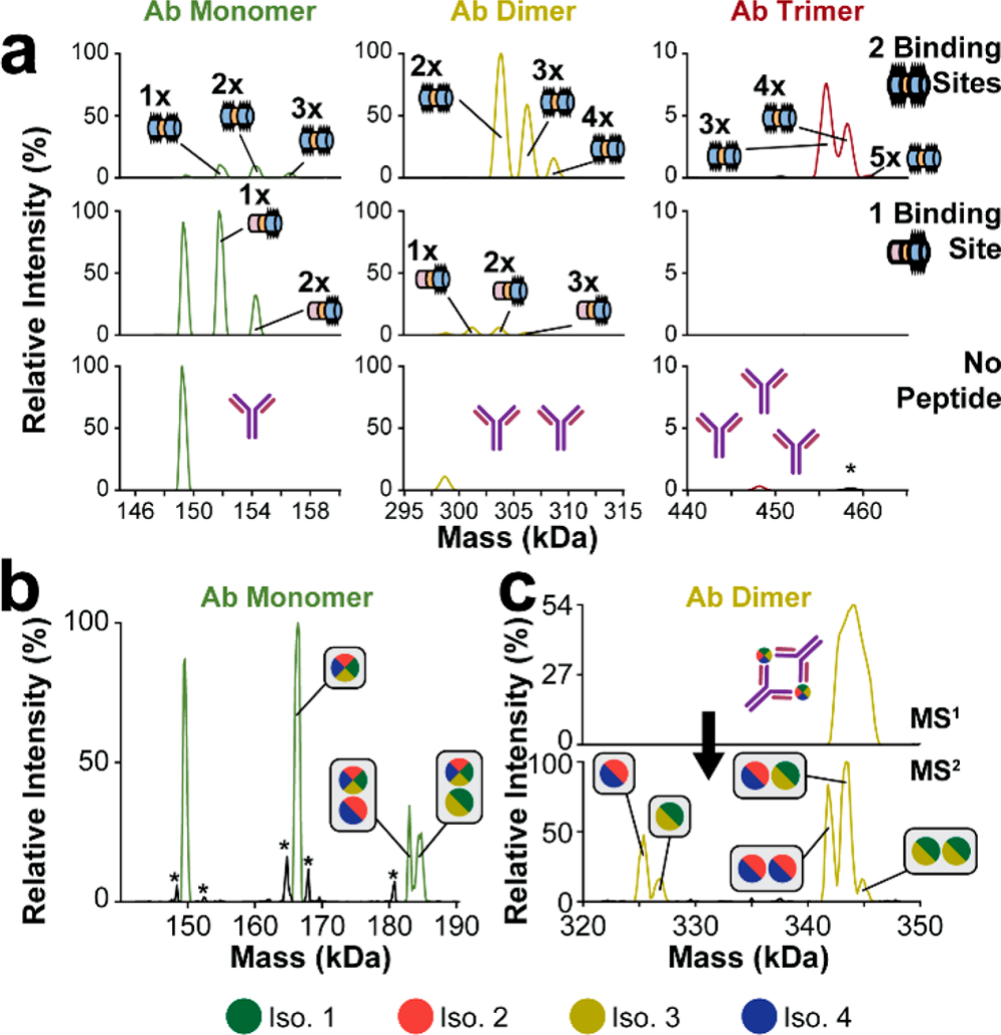
Deconvolved mass spectra of components and complexes (see the detailed experimental methods in the Supporting Information). (a) Measuring antibody–antigen interactions using a two-site peptide (top), a one-site peptide (middle), and a control experiment with no peptide (bottom) (Figure S2). The antibody is measured quantitatively in its monomeric (left), dimeric (center), and trimeric (right) forms. Cartoons indicate how many peptides are bound to the indicated form. Asterisks denote deconvolution artifacts. Peptide colors denote sequence similarities to and distinctions from the two-site region shared among all Ara h 2 isoforms as illustrated in Figures 1 and 3. The antibody is also measured in its (b) monomeric and (c) dimeric forms in complex with the natural Ara h 2 antigen (Figure S5). The dimer is depicted in both unactivated (top) and activated (bottom) schemes. Colored circles denote isoform identity, and multicolored circles denoate isoform ambiguity. Here, the instrument configuration prioritized high-mass transmission, leaving the distribution of ejected antigens unknown.

To probe the interactions between the antibody and antigen (Figure 2a, Table S1), we subjected synthetic peptide antigens bound to a convergent set of IgE complementarity-determining region sequences, presented using an IgG1 scaffold (Table S1, Figure S1), to native MS on a Q Exactive ultra-high-mass range mass spectrometer (Table S2). The basic peptide contains a sequence with two binding sites (“two-site peptides”), known to be conserved across all four Ara h 2 isoforms. A detailed experimental methods section is found in the Supporting Information. An 8.5 μM:10 μM mixture of two-site peptides with the antibody (Figure 2a, top) primarily exhibited masses corresponding to antibody dimerization (85% of the total integrated peak area as deconvolved by UniDec, Table S3)^15^ with some trimerization (8.3%). Two-site peptides bound to the antibody monomer at stoichiometries of 0 (5.0%), 1 (40%), 2 (41%), and 3 (14%), and the peptides also bound to the antibody dimer in stoichiometries of 2 (60%), 3 (33%), and 4 (7.7%). The antibody trimer exhibited two-site peptide binding stoichiometries of 3 (63%), 4 (36%), and 5 (1.1%). We hypothesized that since the antibody dimers bound to a minimum of two peptides as opposed to zero peptides in the monomeric case, the peptides may act as a mediator for antibody dimerization. Otherwise, lower peptide stoichiometries would be prominent in the antibody dimer.

To probe peptide-mediated dimerization, we utilized a version of the peptide where one of the antibody binding sites was replaced by a random sequence, leaving a single binding site on a peptide of comparable length (“one-site peptides”). A one-site peptide experiment exhibited 88% monomeric antibody form and 12% dimerization (Figure 2a, middle). Additionally, in a control experiment with no peptide (Figure 2a, bottom), the antibody exhibited low levels of self-dimerization (18%) and self-trimerization (0.73%) at a 10 μM concentration. The stark contrast between the high level of dimerization in the two-site peptide experiment and the low level of dimerization in both the one-site peptide experiment and the negative control shows that dimerization is strongly dependent on the presence of two antibody binding sites.

The antibody dimerization exhibited during the two-site peptide experiment was antigen-mediated. In addition to the discrepancy in dimer abundance, the highest-abundance charge state of the antibody dimer in the two-site peptide experiment (37+) was higher than in the one-site peptide experiment (34+) and the control (34+) (Figure S2), demonstrating different conformations and, therefore, modes of dimerization (*e.g.*, nonspecific versus antigen-mediated).^16−18^ These data also show that all four Ara h 2 isoforms may be capable of mediating antibody dimerization, as the sequence containing both binding sites in the two-site peptide is conserved across all four isoforms. The two binding sites are separated by a single glutamine residue; therefore, their sequence proximity does not appear to be a barrier for multiple binding events. In other words, the peptide-mediated dimerization predicts antigen-mediated dimerization for all four isoforms given that the protein’s tertiary structure does not obstruct binding regions. As antibody dimers in the two-site peptide experiment only exhibited peptide stoichiometries of 2, 3, and 4, antigen-mediated complexation coincides with both binding regions of at least one antibody being occupied. While some stoichiometries exceed the number of available binding sites in an antigen-mediated model, such as three peptides on the antibody monomer or four peptides on the antibody dimer, these less frequent cases are likely due to nonspecific binding as shown in a “zero-site” peptide experiment (Figure S3). Therefore, most antibody complexation is site-directed, reinforcing the unique dimerization of the two-site experiment.

While the synthetic peptide data predict that all four Ara h 2 isoforms can mediate antibody dimerization, we sought to prove this by probing immunocomplexes containing the Ara h 2 antigens (Figure S4). We mixed Ara h 2 purified from peanut flour and with the IgE construct described above (Table S1) in a 1:1 ratio; this yielded both antibody monomers (81% of the total area, Figure 2b) and antibody dimers (19%; Figure 2c, top). The antigen bound to antibody monomers in stoichiometries of 0 (35%), 1 (43%), and 2 (22%). The two-antigen antibody monomer exhibited two resolved peaks corresponding to an additional light isoform (55%, isoforms 2 and 4) or an additional heavy isoform (45%, isoforms 1 and 3). Low-resolution charge deconvolution of the antigen dimer reveals that the entire antibody dimer distribution was comprised of two antibodies and two unknown antigens. To conduct the study at higher resolution (2×), the complex was isolated and activated using background gas (higher energy collision dissociation, or HCD) (Figure 2c, bottom). The activation removed nonspecific adducts such that the light and heavy isoform groups could be resolved. Activation also yielded a secondary distribution (23% of the total area) of antibody dimers at a higher mass-to-charge ratio (*m*/*z*) than the primary distribution (77%). This secondary distribution is comprised of two antibodies and a single antigen, primarily a light isoform (74%). Most of the two-antigen complexes in the activated spectrum included one heavy isoform and one light isoform (53%), followed by two light isoforms (40%) and two heavy isoforms (7.8%). Despite the improved spectral clarity, the mass differences among isoforms within the same weight group were typically not resolved. The 34+ charge state, for example, did have partially resolved peaks for all four isoforms (Figure S5), but this resolution was not enough to provide satisfactory deconvolution. This experiment demonstrates that intact native MS is insufficient for deep characterization with even modest levels of antigen heterogeneity. However, the secondary distribution in the activated spectrum indicates that HCD had ejected a single antigen that carried away a disproportionate amount of charge (“asymmetric charge partitioning”).^19−21^ This trend validates that antigen ejection and subsequent characterization are possible, which would provide a direct and unambiguous link between antibody and antigen.

**Figure 3:**
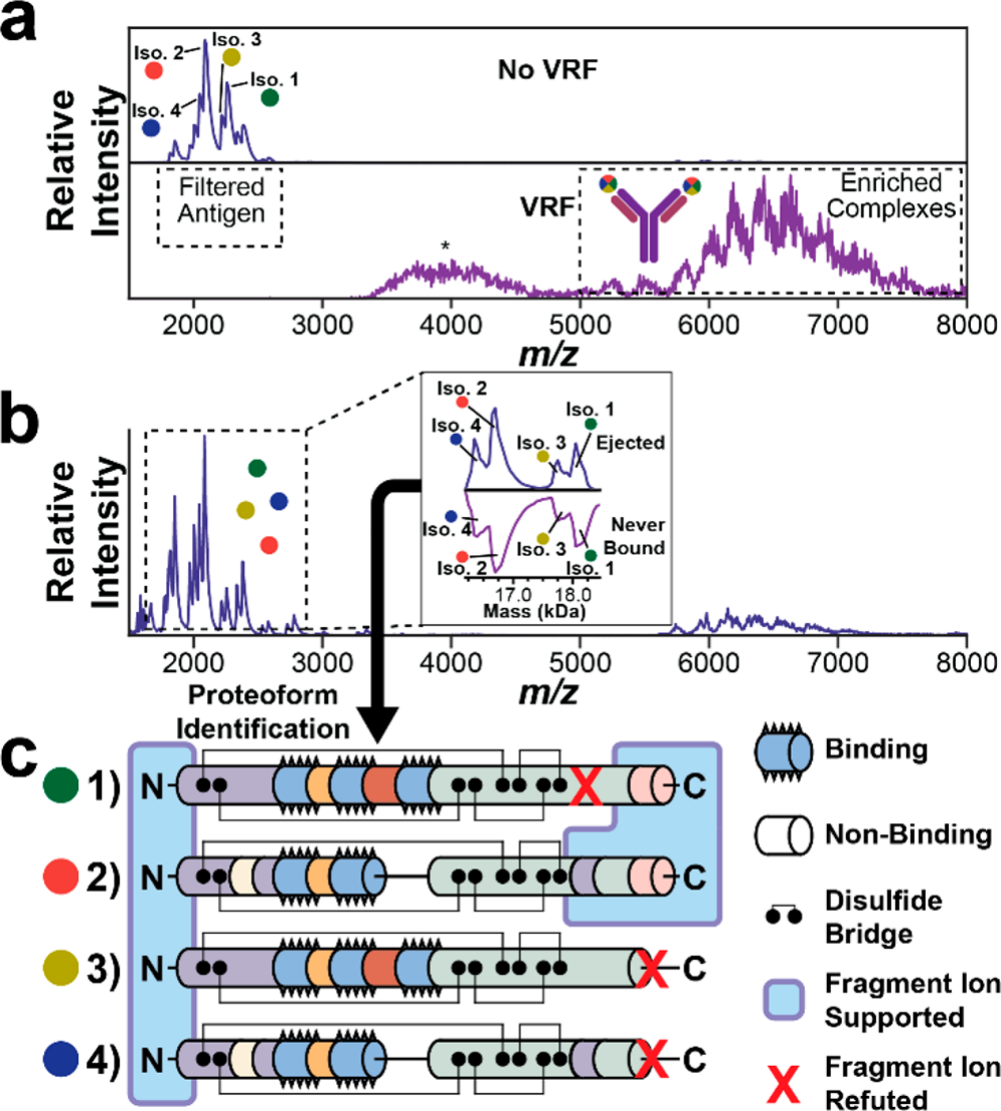
(a) Filtering out of unbound antigens in a complex mixture (top) using voltage rollercoaster filtering (VRF, bottom). Multicolored circles denote isoform ambiguity, and an asterisk denotes an unknown feature that is removed in the subsequent ion trap isolation step. (b) The filtered immunocomplex is activated to eject all proteoforms of bound antigens. The inset shows the deconvolved mass spectrum of the ejected antigen in comparison to that of the antigen that was filtered out using VRF. (c) Depictions of the four Ara h 2 isoforms as well as what regions are supported through the detected fragment ions of isoform 2 (Figure S6).

We next conducted native TDMS on an Orbitrap Eclipse to controllably disassemble the immunocomplex (Figure 3), which was overloaded with the antigen (∼10:1). In this regime, we previously developed a technique called voltage rollercoaster filtering^22,23^ (“VRF”) that markedly enhances the signals of high-mass complexes while simultaneously filtering out unwanted low-mass components such as unbound antigens. With VRF, ions are accelerated into and decelerated out of a high-pressure region near the inlet of the instrument. Unlike their complexed counterparts, unbound antigens lose too much of their kinetic energy through collisions in the high-pressure region to proceed, giving rise to a filtering effect far before the excess antigens can substantially interfere with the complexes via space charge effects. Without VRF, all four isoforms were represented (25% isoform 1, 39% isoform 2, 14% isoform 3, and 23% isoform 4), and the immunocomplex was detected at <10% spectral intensity (Figure 3a, top). After VRF was applied, no unbound antigen was detected, and the monomeric antibody complexes were preserved (Figure 3a, bottom). Then, we used the ion trap to isolate the immunocomplex before HCD. All four isoforms were ejected (22% isoform 1, 41% isoform 2, 15% isoform 3, 22% isoform 4), and the ejected distribution’s similarity to the distribution of the excess unbound antigens suggests that the four isoforms have comparable binding affinities (Figure 3b, including inset). Finally, after isolating a specific proteoform in the ion trap (i.e., isoform 2), the antigen was subjected to further dissociation by HCD (Figure 3c). Disulfide bridges span Ara h 2, preventing complete fragmentation and sequencing; however, the dense residue-by-residue fragmentation on the C-terminus and outside the disulfide bridges was sufficient to distinguish the target. Importantly, ProSight Lite reported a *P*-score of 4.7 × 10^–18^ for these fragmentation data (Figure S6) showing proof-of-concept for confident antigen identification.^24^ The masses of all C-terminal fragment ions support a slightly elongated terminus, consistent with the two additional residues present in isoforms 1 and 2. Furthermore, the C-terminal fragment ions localized a 14 Da loss to E130 (Figure S6), which is consistent with the E130 → D130 substitution detected exclusively in isoforms 2 and 4 (Figure S4). The ability to distinguish isoform 2 from three other isoforms of similar mass directly from the antibody–antigen complex solidifies native TDMS as a viable method for the precise characterization of the immune response.

## Conclusion

This native MS proof-of-concept establishes a basis with which native TDMS can be used to inform our understanding of the immune response. The power in this approach lies in the ability to characterize both the stoichiometry of immunocomplexes and the ejected antigens, whose subtle mass differences typically cannot be resolved by gel- or fluorescence-based readouts. Immunoglobulin therapeutics can be evaluated against the antigens to which they were designed to target as well as disrupters of those immunocomplexes. Differences between the antigens actually bound and the bulk antigen population can reveal how and to what extent a therapeutic can bind—even in complex mixtures.^25^ Whether the antibody consists of a convergent variable region on a monoclonal IgG scaffold (as was done here) or a polyclonal population, the profile of ejected antigens reveals which of their proteoforms are recognized by immunocapture reagent(s). The ability to directly link antigens to the immune response with proteoform-level resolution can reveal how specific functions are triggered in the adaptive immune response including aberrant recognition of endogenous proteins underlying a diverse set of autoimmune disorders.

## Supporting Information Available

Detailed experimental methods, protein sequences, instrument and software parameters, LC-MS data, and complementary native top-down spectra (PDF)
