# Supplementary material for: Immunocomplexed Antigen Capture and Identification by Native Top-Down Mass Spectrometry
Source: J Am Soc Mass Spectrom. 2023 Sep 8;34(10):2093–7. doi: 10.1021/jasms.3c00235 (PMC10557138; doi:10.1021/jasms.3c00235)
Supplement: Supplementary file 1 — js3c00235_si_001.pdf [file js3c00235_si_001.pdf]

Supporting Information

Immunocomplexed Antigen Capture and Identification by Native Top-down Mass Spectrometry

John P. McGee,[a] Rafael D. Melani,[a,†] Ben Des Soye,[a] Derek Croote,[b,†] Valerie Winton,[a,†] Stephen R. Quake,[b,c] Jared O. Kafader,[a] and Neil L. Kelleher[a]*

[a]Departments of Chemistry and Molecular Biosciences and the Proteomics Center of Excellence at Northwestern University, Evanston, IL, 60208; [b]Department of Bioengineering at Stanford University, Stanford, CA, 94305; [c]Department of Applied Physics at Stanford University, Stanford, CA, 94305.

CONTENTS

Detailed Experimental Methods **2**

Table S1. Custom Analyte Sequences **3**

Table S2. UHMR Parameters of Interest **4**

Table S3. UniDec Parameters **5**

Figure S1. Middle-Down LC-MS Characterization of the Utilized Antibody **6**

Figure S2. Peptide-Mediated Dimerization in the *m/z* Domain **7**

Figure S3. 0-Site Peptide-Antibody Experiment **8**

Figure S4. LC-MS Characterization of Ara h 2 .... **9**

Figure S5. Antigen-Mediated Dimerization in the *m/z* Domain **10**

Figure S6. Fragmentation of Isoform 2 .... **11**

References **12**

DETAILED EXPERIMENTAL METHODS

Sample Preparation

Three custom Ara h 2 peptides (sequence on Table S1) were produced and isolated (≥96% purity) by GenScript to test the possible antigen-binding sites. The 2-site peptide represents the isoform 1 sequence from amino acid 59 to 79 (Uniprot Q6PSU2). The sequence in isoform 1 from amino acid 61 to 76 is conserved in all four isoforms and contains two binding sites. The peptides were dissolved in ammonium acetate. Peptides and antibodies were mixed together with a final concentration of 8.5 µM peptide and 10 µM antibody. Samples were incubated on ice for at least 2 min before native TDMS analysis.

For native analysis involving the antibody-antigen complex, the isolated Ara h 2 antigen was acquired from Indoor Biotechnologies (NA-AH2-1). A custom antibody previously described[1] containing the variable region of an IgE and expressed on an IgG scaffold (sequence on Table S1) was recombinantly produced by GenScript. Both proteins were desalted 10 times at 10,000 × *g* for 2-10 minutes in 3 kDa and 100 kDa Amicon Ultra centrifugal filters (Merck Millipore), respectively, with 100 mM ammonium acetate. The antibody was combined with the antigen for a final concentration of 10 µM antigen and 10 µM antibody. The mixture was incubated on ice for at least 2 min before native TDMS analysis.

For middle-down analysis, 20 µg of the antibody construction was digested with IdeS protease (Promega) following the manufacturer's protocol. After digestion, the samples were reduced and cleaned, according to Melani et al. 2019 before LC-MS/MS analysis.[2]

Intact Ara h 2 was also analyzed by LC-MS/MS. Reduction of 20 ug of protein was carried out using 8 M urea and 150 mM TCEP for 1 hour at 37°C. Following the manufacture protocol, the sample was desalted using TopTip C4 (PolyLC). The eluate was dried out using a vacuum concentrator and resuspended in buffer A (94.8% water, 5% acetonitrile, and 0.2% formic acid).

Data Acquisition

Middle-down antibody and intact antigen LC-MS/MS measurements were performed under denaturing conditions using an Ultimate 3000 nanoLC (Thermo Fisher Scientific) equipped with a homemade PLRP-S (5 µm particle size and 1000 Å pore size, Agilent Technologies) trap (30 mm L, 150 µm i.d.) and column (200 mm L, 75 µm i.d.) online with an Orbitrap Fusion Lumos (Thermo Fisher Scientific). The loading pump was operated at 3 µL/min, and the nano-pump gradient flow rate was set at 300 nL/min. LC separation was performed under the following gradient of buffer B (94.8% acetonitrile, 5% water, and 0.2% formic acid): 5% B from 0 to 10 min., 15% B at 13 min., 50% B at 45 min., 95% B from 47 to 49 min., 5% B from 52 to 60 min. The mass spectrometer was operated in "protein mode" with 2 m Torr of N2 pressure, and the acquisition was performed using a data-dependent top 2 MS^2 method. Spray voltage was set at 1,800 V, transfer capillary temperature was set at 320°C, ion funnel RF was set at 30%, and a 15 eV of source CID was applied. MS^1 spectra were acquired at 120,000 of resolving power (at 200 *m/z*), AGC target value of 5×10^5 charges/acquisition, 100 ms. of maximum injection time, and 4 µscans. MS^2 used 23 of NCE for HCD and spectra were acquired at 60,000 resolving power (at 200 *m/z*), with target AGC values of 1×10^6 charges/acquisition, 800 msec of maximum injection time, and 4 µscans. Precursors were quadrupole isolated used using a 3 Th isolation window, dynamic exclusion of 60 sec duration, and threshold of 2×10^4 intensity. Samples were analyzed in technical triplicate.

For native experiments, samples were sprayed via static spray using a Thermo Fisher Nanospray Flex source and medium-sized borosilicate-coated emitters. All studies concerning native antibody dimerization (using the Ara h 2 peptides or the intact antigen) were conducted on a Q Exactive Ultra-High Mass Range (UHMR) instrument from Thermo Fisher Scientific using the parameters listed in Table S2. Data was collected at the highest resolution possible while still allowing annotation: 2-4k resolution.

All studies concerning native antigen-antibody ejection were conducted on an Orbitrap Eclipse (Thermo Fisher) with an HCD collision pressure of 8-20 mTorr in protein mode. Data were collected with a spray voltage of 2-2.5 kV, 2-3 microscans, 120,000 resolving power, 250-5000 ms maximum injection time, and up to 100 averaged scans. Voltage Rollercoaster Filtering was used to filter out unbound antigen, using low levels of activation (20 V IS-CID) and optimized parameters found previously for antibodies.[3] After a broadband ion trap isolation of all complexes, the antigen was ejected using 40 NCE (using mode charge state 25+) for HCD, and the antigen was fragmented using 50 NCE after ion trap of solely isoform 2, charge state 8+. For these experiments, the antibody was at 5 µM, and the antigen was at 50 µM. Data was collected in full profile mode at 120k resolution with 2 microscans and an average of 100 acquisitions. Intact native (UHMR) and native top-down (Eclipse) experiments were repeated to validate the trends, challenges, and methods discussed in the main text, and these repeat experiments yielded comparable results to those reported here.

Data Analysis

Low-resolution (non-isotopically resolved) spectra were deconvolved using UniDec[4] using parameters that minimized spectral artifacts. All used parameters are listed in Table S3 according to the final spectra. Fragmentation data were manually annotated using MMass (v. 5.5.0)[5] and validated using ProSight Lite v.1.4 (10 ppm)[6] on a deconvolved spectrum using Xtract (SN 7, Qual Browser v.4.1.31.9). Annotations were made in reference to UniProt accession Q6PSU2.

Table S1. Custom Analyte Sequences.

Analyte	Sequence
0-Site Peptide	SYGRRELRNLQRELRNLQDPD
1-Site Peptide	SYGRRELRNLQDPYSPSQDPD
2-Site Peptide	SYGRDPYSPSQDPYSPSQDPD
Antibody Light Chain	EIVLTQSPGTLSLSPGGRGTLSCRTSQTINNAHLAWYQHKPGQ APRLLIYGSSERATGVPDRFSGSGSGSDFTLTISSLEAEDFAVY YCQHYGRSPPYTFGPGTKLDIKRTVAAPSVFIFPPSDEQLKSGT ASVVCLLNNFYPREAKVQWKVDNALQSGNSQESVTEQDSKD STYSLSSTLTLSKADYEKHKVYACEVTHQGLSSPVTKSFNRG EC
Antibody Heavy Chain	QVQLVNSGGGVVQPGRSLRLSCVASGFTFSTFGIHWVRQAPG KGLEWVAVISNDGEKSESADSVKGRFTPSRDNSKNTVYLQM NNLRVEDTAVYYCAKVLDYSNYYYYYGMDVWGQGTTVIVS SASTKGPSVFPLAPSSKSTSGGTAALGCLVKDYFPEPVTVSWN SGALTSGVHTFPAVLQSSGLYSLSSVVTVPSSSLGTQTYICNV NHKPSNTKVDKKVEPKSCDKTHTCPPCPAPELLGGPSVFLFPP KPKDTLMISRTPEVTCVVVDVSHEDPEVKFNWYVDGVEVHN AKTKPREEQYNSTYRVVSVLTVLHQDWLNGKEYKCKVSNK ALPAPIEKTISKAKGQPREPQVYTLPPSRDELTKNQVSLTCLV KGFYPSDIAVEWESNGQPENNYKTTPPVLDSDGSFFLYSKLTV DKSRWQQGNVFSCSVMHEALHNHYTQKSLSLSPG

Table S2. Settings of Interest on the Q Exactive UHMR Instrument.

Parameter	Peptide-Antibody Complex	Antigen-Antibody Complex
Nominal Resolution	2188	2188 - 4375
S-lens RF Level	200	200
Ion Transfer Target *m/z*	High	High
Extended Trapping (V)	100	N/A
Normalized Collisional Energy	N/A	0 - 4
Normalization Factor	N/A	1
Ultra High Vacuum Pressure (mbar)	2.21e-10 - 2.25e-10	1.90e-10 - 3.25e-10
Injection Time (ms)	100	400
Detector *m/z*	High	High
Averaging	20	20-26
Microscans	10	20-30
UHMR Mode	On	On

Table S3. UniDec Parameters.

Parameter	Control	0-Site Peptide	1-Site Peptide	2-Site Peptide	Complex MS[1]	Complex MS[2]	Unbound Antigen	Ejected Antigen
**Charge Range**	10-50	20-50	10-50	10-50	20-50	20-50	1-20	1-20
**Mass Range (kDa)**	100-500	100-500	100-500	100-500	100-350	310-340	16.2-18.5	16.2-18.5
**Sample Mass Interval (Da)**	10	10	10	10	200	200	50	50
**Smooth Charge States Distribution**	True	True	True	True	True	True	True	True
**Use Automatic m/z Peak Width**	False	False	False	False	False	False	False	False
**Smooth Nearby Points**	Some	Some	Some	Some	Lots	None	Some	None
**Suppress Artifacts**	Some	Some	Some	Some	Some	Some	Some	Some
**Peak Detection Range (Da)**	1200	1200	1200	1200	300	300	150	150
**Peak Detection Threshold**	0.001	0.001	0.001	0.001	0.1	0.1	0.01	0.25

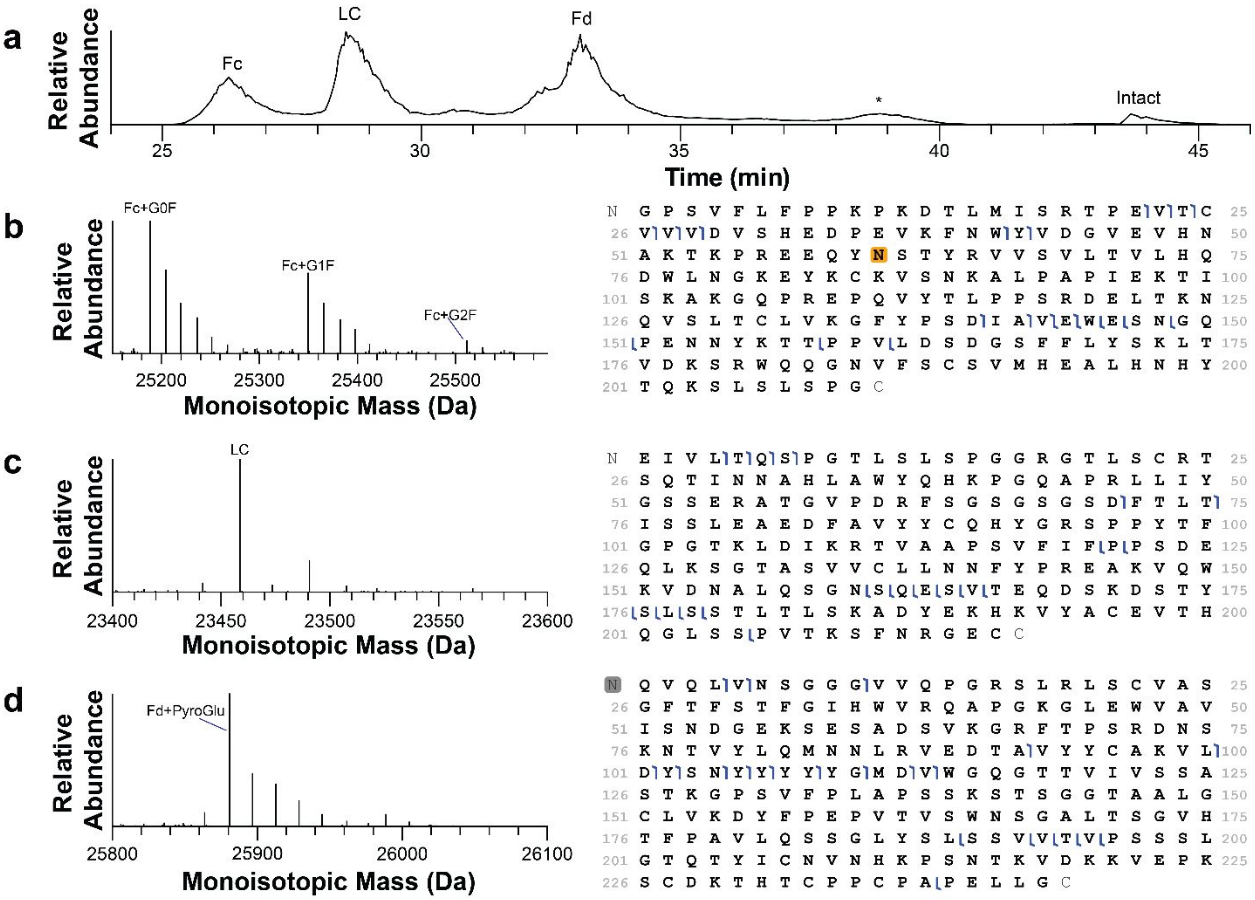

Figure S1. Middle-Down LC-MS Characterization of the Utilized Antibody. (a) Total ion current chromatogram from an LC-MS/MS run of the digested and reduced anti-Ara h 2 antibody. The asterisk denotes an off-target species. (b-d) Deconvolved monoisotopic mass spectra (left) and annotated fragmentation maps (right) of the antibody (b) Fc region, (c) light chain (LC), and (d) Fd region. Orange highlighting on the fragmentation map denotes G0F glycosylation, and grey highlighting denotes the presence of pyroglutamic acid.

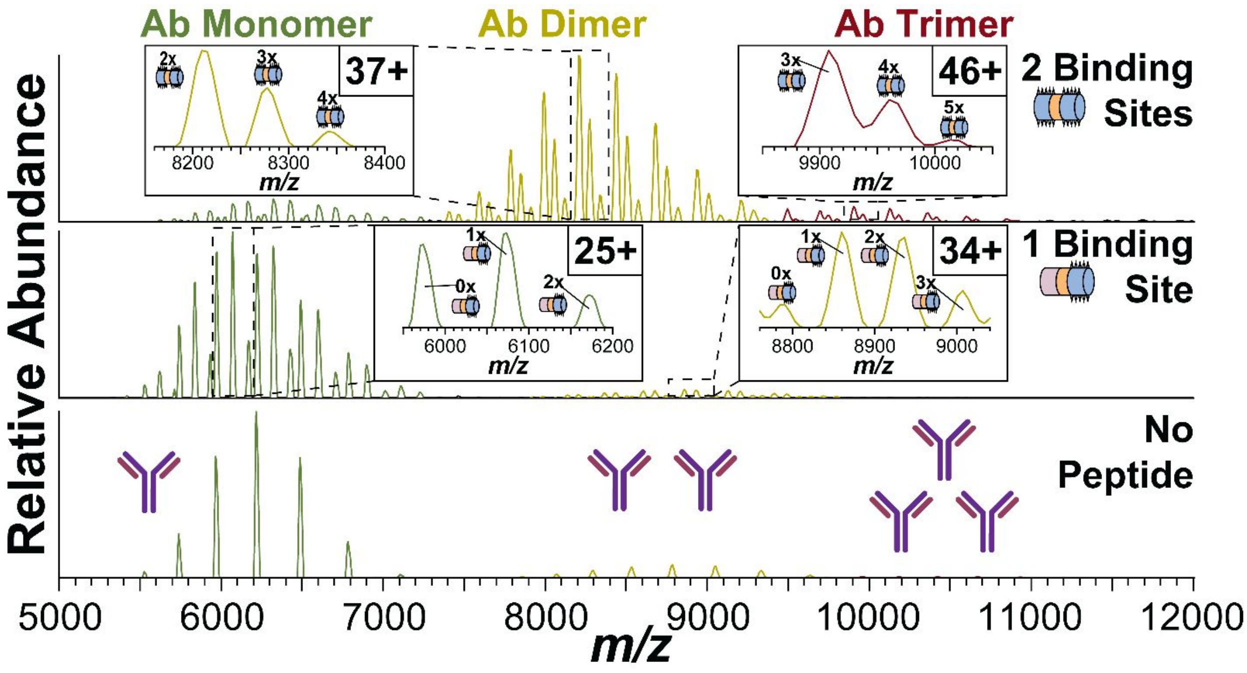

Figure S2. Peptide-Mediated Dimerization in the *m/z* Domain. Measuring antigen-antibody interactions using a 2-site peptide (top), a 1-site peptide (middle), and a control experiment with no peptide (bottom). The antibody is detected in its monomeric (leftmost, green), dimeric (center, yellow), and trimeric (rightmost, red) forms, which are separated in mass-to-charge (*m/z*) space. Insets show select immunocomplex distributions in all three antibody stoichiometries. Cartoons paired with numbers indicate stoichiometries of which type of peptide is present.

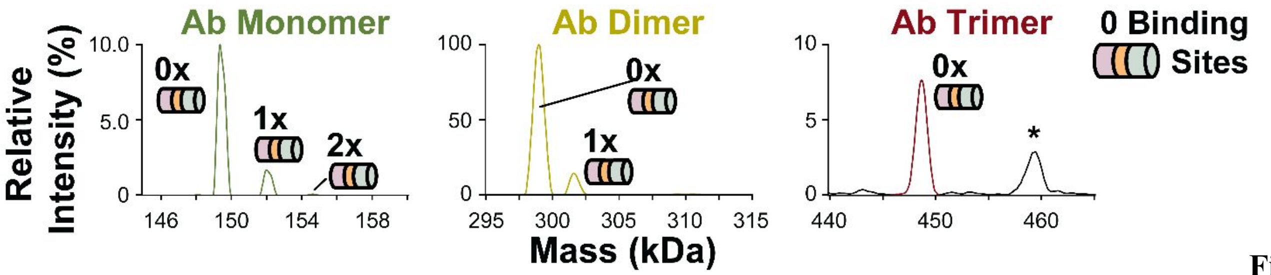

Figure

Figure S3. 0-Site Peptide-Antibody Experiment. Measuring antigen-antibody interactions with a 0-site control peptide. The antibody is measured in its monomeric (left), dimeric (right), and trimeric (right) forms. Cartoons of peptides indicate how many peptides are bound to the indicated form.

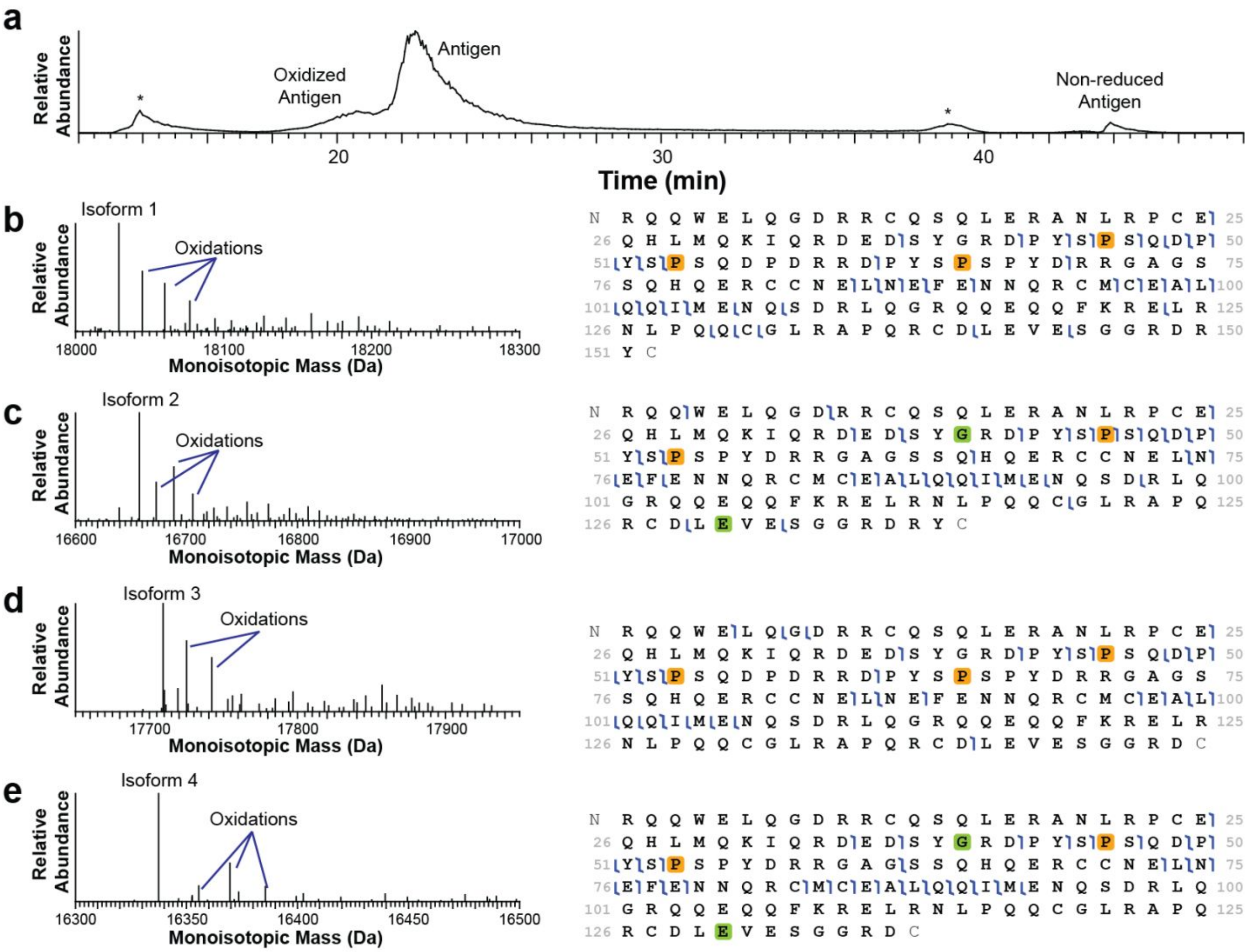

Figure S4. LC-MS Characterization of Ara h 2. (a) The chromatogram for an LC-MS run of the reduced Ara h 2 antigen. Asterisks denote off-target species. (b-e) Deconvolved monoisotopic mass spectra (left) and annotated fragmentation maps (right) of Ara h 2 (b) Isoform 1, (c) Isoform 2, (d) Isoform 3, and (e) Isoform 4. Orange highlights on the fragmentation maps denote hydroxyprolines, and green highlights on the fragmentation maps denote substitutions G40→E40 and E130→D130.

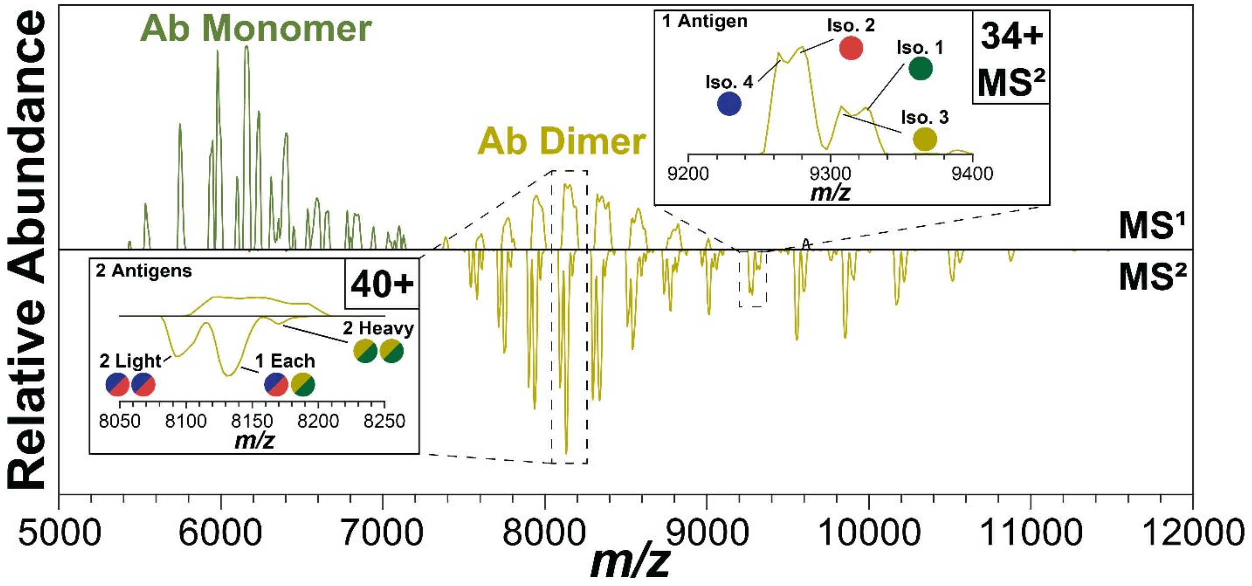

Figure S5. Antigen-Mediated Dimerization in the *m/z* Domain. Measuring antigen-antibody interactions using the natural Ara h 2 antigen with the antibody, depicted in the mass-to-charge (*m/z*) space. The antibody is measured in its monomeric (leftmost, green) and dimeric (rightmost, yellow) forms. The dimer is depicted in both unactivated (top) and activated (bottom) schemes. Insets show select immunocomplex distributions at specified charge states. Colored dots indicate clarity or ambiguity in the annotation of antigen stoichiometries.

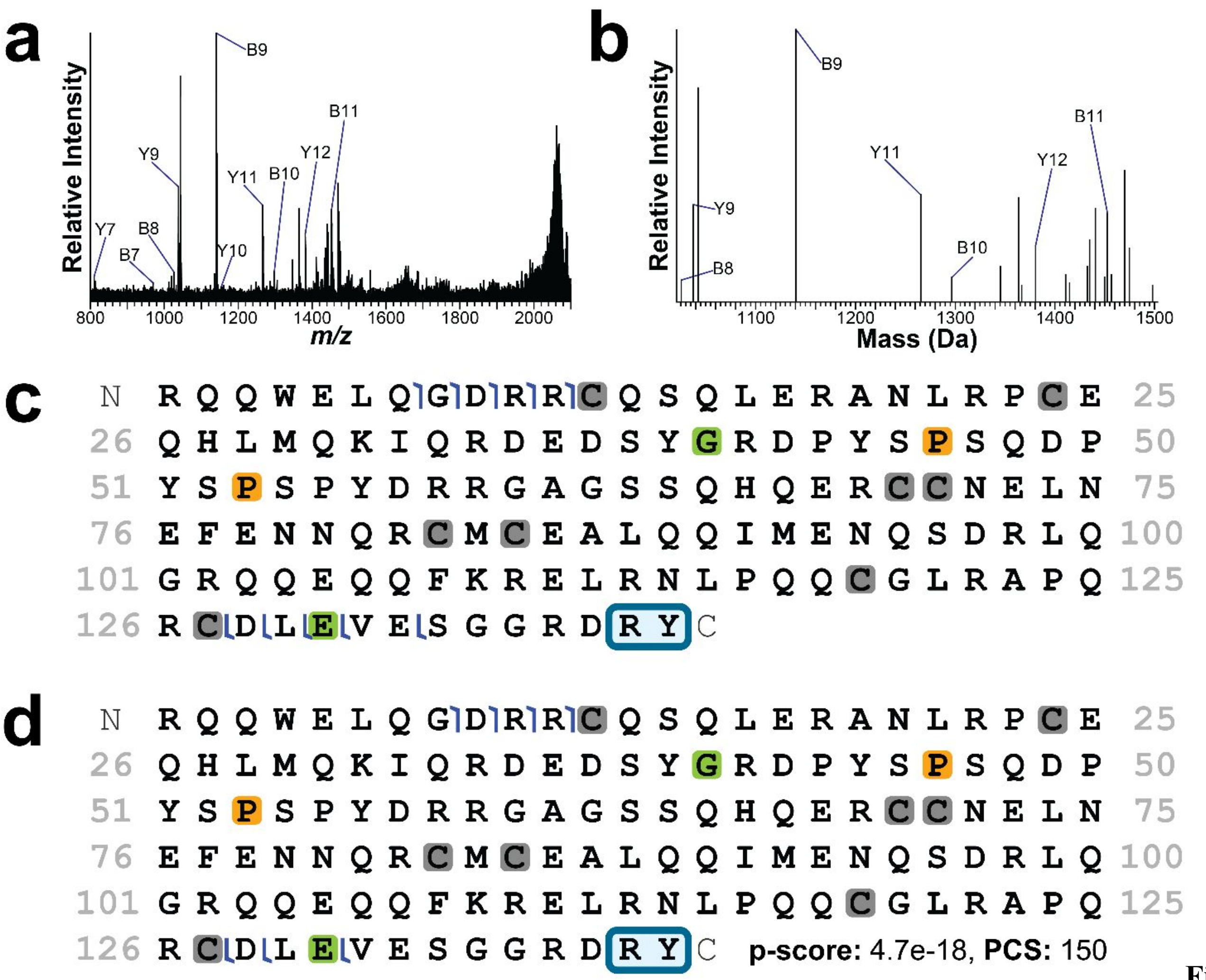

Figure

Figure S6. Fragmentation of Isoform 2. (a) Fragmentation spectrum and (b) deconvolved mass spectrum of natural Ara h 2 Isoform 2 in a native top-down mass spectrometry experiment of the immunocomplex. (c) Manually annotated and (d) ProSight Lite annotated fragmentation maps of the data. Each blue flag indicates a detected and annotated fragment ion that contains the region extending from the flag to the terminus towards which the flag points. Grey highlights indicate the location of one-half of a disulfide bridge. Orange highlights indicate the presence of hydroxyprolines. Green highlights indicate the presence of substitutions G40→E40 and E130→D130, present on isoforms 2 and 4. The blue highlight indicates a region conserved in isoforms 1 and 2 of the antigen.

REFERENCES

(1) Croote, D.; Darmanis, S.; Nadeau, K. C.; Quake, S. R. High-affinity allergen-specific human antibodies cloned from single IgE B cell transcriptomes. *Science* **2018**, *362*, 1306.

(2) Melani, R. D.; Srzentić, K.; Gerbasi, V. R.; McGee, J. P.; Huguet, R.; Fornelli, L.; Kelleher, N. L. Direct measurement of light and heavy antibody chains using ion mobility and middle-down mass spectrometry. *mAbs* **2019**, *11*, 1351-1357.

(3) McGee, J. P.; Melani, R. D.; Goodwin, M.; McAlister, G.; Huguet, R.; Senko, M. W.; Compton, P. D.; Kelleher, N. L. Voltage Rollercoaster Filtering of Low-Mass Contaminants During Native Protein Analysis. *J. Am. Soc. Mass Spectrom.* **2020**, *31*, 763-767.

(4) Marty, M. T.; Baldwin, A. J.; Marklund, E. G.; Hochberg, G. K.; Benesch, J. L.; Robinson, C. V. Bayesian deconvolution of mass and ion mobility spectra: from binary interactions to polydisperse ensembles. *Anal. Chem.* **2015**, *87*, 4370-4376.

(5) Strohalm, M.; Kavan, D.; Novák, P.; Volný, M.; Havlíček, V. mMass 3: A Cross-Platform Software Environment for Precise Analysis of Mass Spectrometric Data. *Anal. Chem.* **2010**, *82*, 4648-4651.

(6) Fellers, R. T.; Greer, J. B.; Early, B. P.; Yu, X.; LeDuc, R. D.; Kelleher, N. L.; Thomas, P. M. ProSight Lite: graphical software to analyze top-down mass spectrometry data. *Proteomics* **2015**, *15*, 1235-1238.
